# Meta-analysis of two Chinese populations identifies an autoimmune disease risk allele in 22q11.21 as associated with systemic lupus erythematosus

**DOI:** 10.1186/s13075-015-0577-6

**Published:** 2015-03-20

**Authors:** Yan Zhang, Yong-Fei Wang, Jing Yang, Jing Zhang, Liangdan Sun, Nattiya Hirankarn, Hai-Feng Pan, Chak Sing Lau, Tak Mao Chan, Tsz Leung Lee, Alexander Moon Ho Leung, Chi Chiu Mok, Lu Zhang, Jiangshan Jane Shen, Sik Nin Wong, Ka Wing Lee, Marco Hok Kung Ho, Pamela Pui Wah Lee, Brian Hon-Yin Chung, Chun Yin Chong, Raymond Woon Sing Wong, Mo Yin Mok, Wilfred Hing Sang Wong, Kwok Lung Tong, Niko Kei Chiu Tse, Xiang-Pei Li, Yingyos Avihingsanon, Pornpimol Rianthavorn, Thavatchai Deekajorndej, Kanya Suphapeetiporn, Vorasuk Shotelersuk, Shirley King Yee Ying, Samuel Ka Shun Fung, Wai Ming Lai, Chun-Ming Wong, Irene Oi Lin Ng, Maria-Merce Garcia-Barcelo, Stacey S Cherny, Paul Kwong-Hang Tam, Pak Chung Sham, Sen Yang, Dong Qing Ye, Yong Cui, Xue-Jun Zhang, Wanling Yang, Yu Lung Lau

**Affiliations:** Department of Paediatrics and Adolescent Medicine, Li Ka Shing Faculty of Medicine and Queen Mary Hospital, The University of Hong Kong, 21 Sassoon Road, Hong Kong, China; State Key Laboratory Incubation Base of Dermatology, Key Laboratory of Dermatology, Anhui Medical University, Ministry of Education, 81 Meishan Road, Hefei, Anhui 230032 China; Lupus Research Unit, Department of Microbiology, Faculty of Medicine, Chulalongkorn University, 254 Phayathai Road, Bangkok, 10330 Thailand; Department of Epidemiology and Biostatistics, School of Public Health, Anhui Medical University, Hefei, Anhui 230032 China; Department of Medicine, Queen Mary Hospital, Li Ka Shing Faculty of Medicine, The University of Hong Kong, 21 Sassoon Road, Hong Kong, China; Department of Medicine, Queen Elizabeth Hospital, 30 Gascoigne Road, Hong Kong, China; Department of Medicine, Tuen Mun Hospital, New Territory, Hong Kong, China; Department of Paediatrics and Adolescent Medicine, Tuen Mun Hospital, 23 Tsing Chung Koon Road, Hong Kong, China; Department of Medicine, Pamela Youde Nethersole Eastern Hospital, 3 Lok Man Road, Hong Kong, China; Department of Medicine, Princess Margaret Hospital, 2-10 Princess Margaret Hospital Road, Hong Kong, China; Department of Paediatrics and Adolescent Medicine, Princess Margaret Hospital, 2-10 Princess Margaret Hospital Road, Hong Kong, China; Department of Rheumatology, Anhui Provincial Hospital, 424 West Changjiang Road, Hefei, China; Department of Medicine, Faculty of Medicine, Chulalongkorn University, 254 Phayathai Road, Bangkok, 10330 Thailand; Department of Pediatrics, Faculty of Medicine, Chulalongkorn University, 254 Phayathai Road, Bangkok, 10330 Thailand; Department of Pathology, Li Ka Shing Faculty of Medicine, The University of Hong Kong, 21 Sassoon Road, Hong Kong, China; Department of Surgery, Li Ka Shing Faculty of Medicine, The University of Hong Kong, 21 Sassoon Road, Hong Kong, China; Department of Psychiatry, Li Ka Shing Faculty of Medicine, The University of Hong Kong, 21 Sassoon Road, Hong Kong, China; Centre for Genomic Sciences, Li Ka Shing Faculty of Medicine, The University of Hong Kong, 21 Sassoon Road, Hong Kong, China

## Abstract

**Introduction:**

Systemic lupus erythematosus (SLE) is a heterogeneous disease with a diverse spectrum of clinical symptoms, ranging from skin rash to end-organ damage. 22q11.21 has been identified as a susceptibility region for several autoimmune diseases, including SLE. However, detailed information for SLE association and the underlying functional mechanism(s) is still lacking.

**Methods:**

Through meta-analysis of two genome-wide association studies (GWAS) on Han Chinese populations, comprising a total of 1,659 cases and 3,398 controls matched geographically, we closely examined the 22q11.21 region, especially on the reported single-nucleotide polymorphisms (SNPs) associated with different autoimmune diseases and their relationships. We further replicated the most significant associations of SNPs with SLE using 2,612 cases and 2,323 controls of Asian ancestry.

**Results:**

All reported SNPs in the 22q11.21 region with different autoimmune diseases were examined using the two GWAS data and meta-analysis results, and supportive evidence of association with SLE was found (meta-analysis: *P*_meta ≤ 7.27E-05), which might require further investigation. SNP rs2298428 was identified as the most significant SNP associated with SLE in this region (*P*_meta =2.70E-09). It showed independent effects through both stepwise and conditional logistic regression, and there is no evidence of other independent association signals for SLE in this region. The association of rs2298428 was further replicated in three cohorts from Hong Kong, Anhui and Thailand comprising a total of 2,612 cases and 2,323 controls (joint analysis of GWAS and replication result: *P*_all =1.31E-11, odds ratio =1.23). SNP rs2298428 was shown to be an expression quantitative locus for *UBE2L3* gene in different cell types, with the risk allele (T) being correlated with higher expression of UBE2L3. This is consistent with earlier reports on higher expression of *UBE2L3* in patients with SLE.

**Conclusions:**

Association with distinct autoimmune diseases highlights the significance of this region in autoreactive responses and potentially shared functional mechanisms in these diseases.

**Electronic supplementary material:**

The online version of this article (doi:10.1186/s13075-015-0577-6) contains supplementary material, which is available to authorized users.

## Introduction

Systemic lupus erythematosus (SLE) is an autoimmune disease with an unclear etiology. It usually presents with a diverse spectrum of clinical manifestation spanning from malar rash to kidney injury. Genetic factors explain about 50% to 60% of the disease etiology [1]. The concordance rate for SLE is much higher in monozygotic (25% to 70%) than in dizygotic (2% to 9%) twins [2,3], indicating the importance of genetic contributions.

In genome-wide association studies (GWAS) on SLE, researchers have identified more than 40 loci associated with the disease [4-16]. Many regions, such as *STAT4*, *BLK* and *IRF5*, were found to modulate risk of multiple diseases, although the causal variant(s) may not be shared by different diseases [17]. Cotsapas *et al*. [17] estimated that 44% of single-nucleotide polymorphisms (SNPs) associated with one autoimmune disease might also be associated with another one. These observations support the hypothesis that autoimmune diseases may share common genetic basis.

The 22q11.21 genomic region was found to be associated with multiple autoimmune diseases, including SLE [14,18], systemic sclerosis (SSc) [19], Crohn’s disease (CD) [20], celiac disease (CeD) and rheumatoid arthritis (RA) [21], psoriasis (PS) [22] and inflammatory bowel disease (IBD) [23]. SNP rs5754217-A (denoting risk allele A of rs5754217), located in the intron of *UBE2L3*, showed a suggestive association with SLE in women of European ancestry (*P* = 7.53 × 10^−8^) [18]. SNP rs463426-A and rs131654-A, located downstream of *HIC2* and upstream of *UBE2L3*, was identified as susceptibility variants with SLE in a Han Chinese population (*P* = 1.48 × 10^−16^ and 2.99 × 10^−16^, respectively) [14]. SNP rs2298428-T, which is a missense variant in *YDJC*, was significantly enriched in diffuse SSc, though no independent study showed the variant reaching GWAS significance (*P* =0.017) [19]. SNP rs2298428-T was also suggested to be associated with CD (*P* = 5.22 × 10^−5^) [20]. The same SNP was also reported to be associated with both CeD and RA (rs2298428-T, *P* = 2.5 × 10^−10^) [21]. Another SNP, rs181359-A, located in intron 2 of *UBE2L3*, was established as associated with PS and CD (*P* = 8.02 × 10^−10^, *P* = 6.3 × 10^−13^, respectively) [22]. SNP rs2266959-T, also located in intron 2 of *UBE2L3*, was also robustly associated with IBD (*P* = 1 × 10^−16^) [23]. Despite the various reports on this region, the details of association and potential independent effects are unclear. Further elucidation of the signals in this region may help improve understanding of the shared etiological basis among autoimmune and inflammatory diseases.

In the present study, we examined the association for SLE in the 22q11.21 region and further replicated the association of SNP rs2298428 in a total of 4,271 cases and 5,721 controls of Asian ancestry. To that end, our results confirmed rs2298428 as the most significant SNP associated with SLE in this region. Meanwhile, the risk allele of this SNP is highly correlated with higher expression of *UBE2L3* in different cell lines.

## Methods

### Study participants

The samples included in the present study were collected from Hong Kong and Anhui, China, and from Bangkok, Thailand (Additional file 1). All the cases fulfilled the revised criteria of the American College of Rheumatology for diagnosis of SLE. Cases from Hong Kong were recruited from five hospitals in Hong Kong: Queen Mary Hospital, Tuen Mun Hospital, Queen Elizabeth Hospital, Pamela Youde Nethersole Eastern Hospital and Princess Margaret Hospital (HK_GWAS and HK_REP). Clinical records were well documented with autoantibody profiles and subphenotypes. Controls from Hong Kong were individuals from other GWAS studies who had no overlapping manifestations with SLE in the discovery stage (HK_GWAS). Cases from Anhui were patients visiting the Department of Rheumatology at Anhui Provincial Hospital and the First Affiliated Hospital of Anhui Medical University in Hefei, Anhui province (AH_REP), with corresponding controls from healthy blood donors in Anhui (AH_REP). The cases for the Anhui GWAS (AH_GWAS) were recruited from several hospitals in central and southern China, and the controls were carefully selected with geographically matched and clinically unrelated individuals (AH_GWAS). The cases from Thailand were patients at King Chulalongkorn Memorial Hospital (TH_REP), and geographically matched healthy donors were used as controls (TH_REP). All the individuals involved in the present study gave informed consent. The study conducted in Hong Kong was approved by the institutional review board of the University of Hong Kong/Hospital Authority Hong Kong West Cluster. The study in Anhui was approved by the institutional review board of Anhui Medical University. The Thailand study was approved by the intuitional review board of the Faculty of Medicine, Chulalongkorn University, Bangkok, Thailand.

### Imputation

IMPUTE2.3.1 was used to perform imputation on the Hong Kong and Anhui data by using all the samples from 1000 Genome Project (released in September 2013). SNPs that violated Hardy-Weinberg equilibrium (HWE) and SNPs with minor allele frequency <0.05% were removed for further analysis.

### Genotyping

The GWAS on the Hong Kong and Anhui cohorts were conducted using the Illumina Human610-Quad BeadChip array (Illumina, San Diego, CA, USA), as previously reported (HK_GWAS and AH_GWAS). Further replication of the candidate SNPs was performed by using the TaqMan genotyping method (Life Technologies, Carlsbad, CA, USA) with the remaining samples from the Hong Kong cohort that were not included in the discovery stage (HK_REP); samples collected from Bangkok, Thailand (TH_REP); and samples from an independent Anhui cohort (AH_REP). Genotyping concordance between Illumina Human610-Quad BeadChip and TaqMan SNP genotyping method was also checked on randomly selected samples, and the two methods showed complete concordance.

### Association analysis

We used inverse variance method for the meta-analysis installed in METAL [24]. Joint analysis of association was conducted using the Cochran-Mantel-Haenszel (CMH) test, taking into account the effect of SNP differences between cohorts. The homogeneity of the effect size between different cohorts and different stages of the study was evaluated by using the Breslow-Day test (*P*_het in Table 1), both installed in PLINK 1.07.

**Table 1:** **Association results of single-nucleotide polymorphism rs2298428 from each cohort and joint analysis** ^**a**^

**SNP**	**Gene/AA change**	**HK (HK_GWAS + HK_REP)**				**AH (AH_GWAS + AH_REP)**				**TH_REP**				**Combination**		
		**F_A**	**F_U**	***P*** **-value**	**OR**	**F_A**	**F_U**	***P*** **-value**	**OR**	**F_A**	**F_U**	***P*** **-value**	**OR**	***P*** **_all**	**OR_all (CMH test)**	***P*** **_het**
rs2298428	*YDJC*/Ala263Thr	0.50	0.46	1.71E-03	1.17	0.44	0.37	3.16E-09	1.29	0.49	0.45	0.06	1.16	1.31E-11	1.23 (1.16 to 1.30)	0.26

^a^AA, Amino acid; AH, Anhui; AH_GWAS + AH_REP, Cases from Anhui were patients visiting the Department of Rheumatology at Anhui Provincial Hospital and the First Affiliated Hospital of Anhui Medical University in Hefei, Anhui province (AH_REP), with corresponding controls from healthy blood donors in Anhui (AH_REP). The cases for the Anhui GWAS (AH_GWAS) were recruited from several hospitals in central and southern China, and the controls were carefully selected with geographically matched and clinically unrelated individuals (AH_GWAS); GWAS, Genome-wide association studies; HK, Hong Kong; HK_GWAS + HK_REP, Cases from five hospitals in Hong Kong: Queen Mary Hospital, Tuen Mun Hospital, Queen Elizabeth Hospital, Pamela Youde Nethersole Eastern Hospital and Princess Margaret Hospital; TH_REP, Cases from Thailand were patients at King Chulalongkorn Memorial Hospital. F_A/F_U indicates minor allele frequency of the single-nucleotide polymorphism (SNP) in cases or controls. The calculation of odds ratio (OR) is also based on the minor allele of each SNP.

Stepwise logistic regression was performed using IBM SPSS 16.0 software (IBM, Armonk, NY, USA). Tests of independent contributions toward disease associations for SNPs in a single locus were done using logistic regression, adjusting for the effect of a specific SNP in the same locus, while also taking into account differences among cohorts. SNPTEST v2.2.0 was used to perform the logistic regression tests in this study [25]. Linkage disequilibrium (LD) patterns and values were obtained using Haploview [26].

## Results

### Imputation and meta-analysis of two genome-wide association studies on Han Chinese populations from Hong Kong and Anhui

First, imputation was performed using IMPUTE2 [27] on two GWAS on Han Chinese populations. Association analysis was performed using SNPTEST v2.2.0, taking the genotype uncertainty into account. Meta-analysis was performed using METAL [24] with the inverse variance-based model. We examined the meta-analysis results in 22q11.21 and observed a total of 4,834 SNPs in this 1.9-Mb region, from 20,220,110 to 22,131,990 bp (GRCh37/hg19). On the basis of meta-analysis *P*-values (*P*_meta), 121 SNPs showed suggestive associations (*P*_meta <0.0001), aggregating in a 187-kb region (Additional file 2). SNP rs2298428 showed the most significant *P*-value (*P*_meta =2.70E-09). Of the 96 SNPs, 25 SNPs had *P*_meta-values reaching genome-wide significance (5E-08), including SNP rs2298428. The other 24 SNPs all had high LD with rs2298428 (*r*^2^ > 0.9).

### Linkage disequilibrium pattern of single-nucleotide polymorphisms in 22q11.21 associated with immune-related diseases

**Figure 1:**
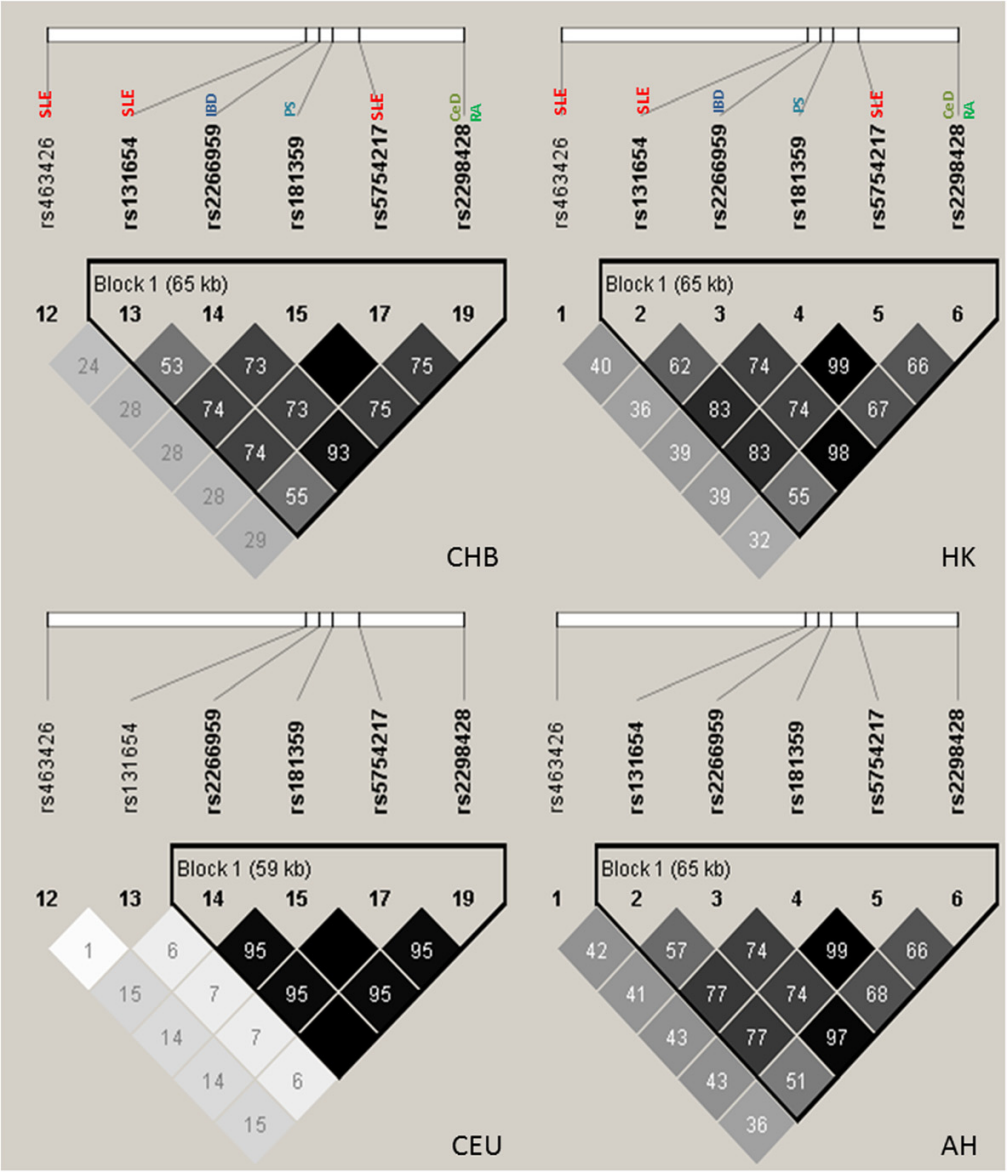
**The linkage disequilibrium patterns of the susceptibility single-nucleotide polymorphisms for autoimmune diseases in different populations.** AH, Anhui; CeD, Celiac disease; CEU, Utah residents with ancestry from northern and western Europe; CHB, Han Chinese, Beijing; HK, Hong Kong; IBD, Inflammatory bowel disease; PS, Psoriasis; RA, Rheumatoid arthritis; SLE, Systemic lupus erythematosus.

For the purpose of finding potentially shared susceptible variants and/or causal variants between SLE and other immune-related diseases, we focused on SNP rs2298428, which showed the most significant *P*-values for SLE in our meta-analysis results, and other reported SNPs in this region that showed association with SLE (rs5754217, rs463426 and rs131654) [14,18], CD and PS (rs181359) [22], and IBD (rs2266959). SNP rs2298428 was also reported to be associated with other immune-related diseases, including SSc [19], CeD and RA [21]. As shown in Table 2, all six SNPs reported for different diseases showed strong evidence of association with SLE in Asian populations (2.70E-09 ≤ − ≤ 7.27E-05). The LD patterns of these six SNPs are shown in Figure 1, based on different populations from HapMap data including Han Chinese, Beijing, population and Utah residents with ancestry from northern and western Europe (CHB and CEU, respectively), Hong Kong (HK) and Anhui (AH). The LD patterns in CHB, HK and AH populations were similar. In these three Chinese populations, all but rs463426 showed moderate to high LD between each other (*r*^2^ > 0.5). We also compared the LD patterns between the Chinese populations and CEU (Figure 1). In Caucasians, SNPs rs463426 and rs131654 showed minimal LD with all the other SNPs (*r*^2^ < 0.2). The other four SNPs showed high LD with each other (*r*^2^ > 0.95).

**Table 2:** **Meta-analysis results for single-nucleotide polymorphisms reported in previous genome-wide association studies on other immune-related diseases in the 22q11.21 region** ^**a**^

**G/I**	**SNP**	**Base pair**	**A1/A2**	**AH**			**HK**			***P*** **_meta-value**	***P*** **_het-value**	**Disease(s)**
				**F_A/F_U**	***P*** **-value**	**OR (95% CI)**	**F_A/F_U**	***P*** **-value**	**OR (95% CI)**			
G	rs463426	21,809,185	C/*T*	0.46/0.53	3.62E-05	0.78 (0.70 to 0.88)	0.42/0.45	0.08	0.89 (0.79 to 1.02)	2.50E-05	0.14	SLE
G	rs131654	21,917,190	G/*T*	0.41/0.48	1.60E-05	0.77 (0.69 to 0.87)	0.38/0.40	0.24	0.93 (0.81 to 1.05)	7.27E-05	0.04	SLE
I	rs2266959	21,922,904	*T*/G	0.45/0.37	3.22E-07	1.37 (1.21 to 1.54)	0.52/0.47	1.66E-03	1.22 (1.07 to 1.38)	4.41E-09	0.27	IBD
I	rs181359	21,928,641	*A*/G	0.53/0.46	1.86E-06	1.33 (1.18 to 1.50)	0.58/0.56	0.07	1.12 (0.99 to 1.28)	2.36E-06	0.06	PS, CD
G	rs5754217	21,939,675	*T*/G	0.53/0.46	1.04E-06	1.34 (1.19 to 1.51)	0.59/0.56	0.09	1.12 (0.98 to 1.27)	2.07E-06	0.04	SLE
G	rs2298428	21,982,892	*T*/C	0.44/0.37	4.72E-07	1.37 (1.21 to 1.54)	0.51/0.46	8.44E-04	1.24 (1.09 to 1.40)	2.70E-09	0.36	CD, CeD, RA, SSc

^a^AH, Anhui; CeD, Celiac disease; CS, Crohn’s disease; CI, Confidence interval; HK, Hong Kong; IBD, Inflammatory bowel disease; PS, Psoriasis; RA, Rheumatoid arthritis; SSc, Systemic sclerosis; SLE, Systemic lupus erythematosus. G/I indicates whether the single-nucleotide polymorphism (SNP) are genotyped (G) or imputed (I). A1/A2 indicates the minor allele/major allele of the SNP. F_A/F_U indicates minor allele frequency of the SNP in cases or controls. The calculation of odds ratio (OR) is also based on the minor allele of each SNP. The risk allele of each SNP is italics.

### Independence test on the single-nucleotide polymorphisms associated with immune-related diseases

A stepwise logistic regression analysis was performed to test the independence of these SNPs (Table 3). The method begins with an empty model, to which the variables were added one at a time. The analysis showed that SNP rs2298428 exhibited the strongest, and the only, significant association with SLE. Further addition of any other SNPs involved did not show significant improvement of the model, which is partially explained by the high LD among most of these SNPs. However, the SNPs with moderate LD did not show significant improvement in the model, demonstrating a lack of evidence of further independent signals of association for SLE in this region.

**Table 3:** **Independent effects among the single-nucleotide polymorphisms in the 22q11.21 region** ^**a**^

**SNP added to the model**	***P*** **-value**	**OR (95% CI)**
rs463426	0.76	–
rs131654	0.47	–
rs2266959	0.46	–
rs181359	0.31	–
rs5754217	0.36	–
rs2298428	2.48E-08	1.29 (1.18 to 1.41)

^a^CI, Confidence interval; OR, Odds ratio; SNP, Single-nucleotide polymorphism.

**Figure 2:**
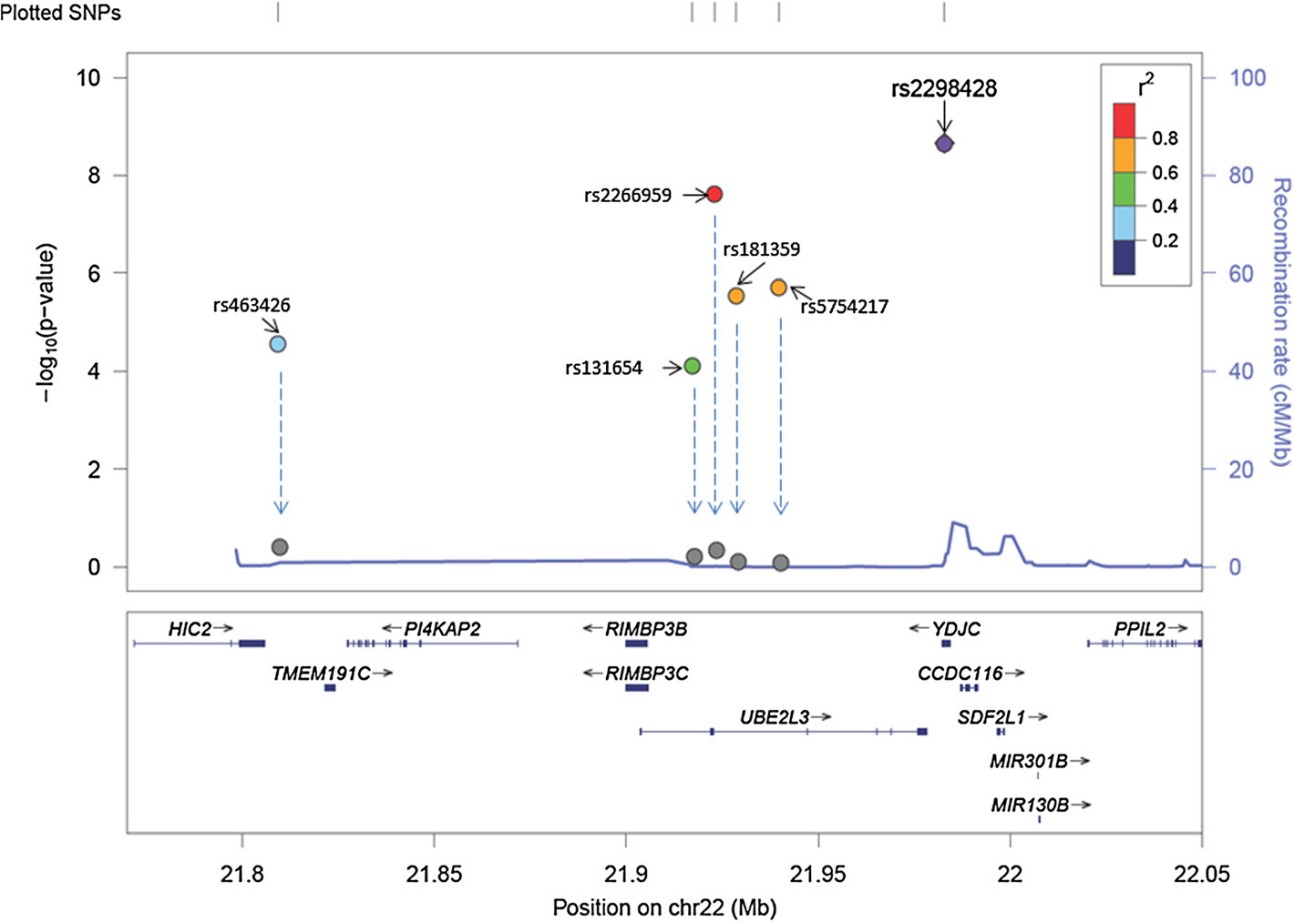
**Association** ***P*** **-values of all single-nucleotide polymorphisms before and after adjustment for the effect of rs2298428.** The graph depicts the association *P* **-**values of all single-nucleotide polymorphisms (SNPs) before (the gradient colors of dots reflects the linkage disequilibrium (LD) between the SNPs and SNP rs2298428, using hg19/1000 Genomes March 2012 ASN (Asian) as a reference) and after (gray) adjustment for the effect of rs2298428. The arrows show the reduction of the SNPs after the association adjusted by the effect of rs2298428.

Conditional logistic regression analysis was performed to investigate the independent effects among these replicated SNPs with immune-related diseases (Table 4). SNP rs2298428 remained significant when the effect of any other SNPs was accounted for, except for SNP rs2266959. More intuitively, the association *P*-values of all SNPs before and after the effect of rs2298428 was adjusted for are shown in Figure 2.

**Table 4:** **Conditional logistic regression analysis** ^**a**^

		**SNP whose effect is adjusted for**					
		**rs463426**	**rs131654**	**rs2266959**	**rs181359**	**rs5754217**	**rs2298428**
rs463426	OR (95% CI)		1.14 (1.01 to 1.27)	1.02 (0.91 to 1.14)	1.1 (0.98 to 1.23)	1.09 (0.98 to 1.23)	1.05 (0.94 to 1.17)
	*P*-value		**0.03**	0.74	0.12	0.12	0.39
rs131654	OR (95% CI)	1.10 (0.98 to 1.24)		0.95 (0.82 to 1.10)	0.99 (0.81 to 1.21)	0.94 (0.77 to 1.15)	0.96 (0.85 to 1.10)
	*P*-value	0.09		0.51	0.92	0.57	0.59
rs2266959	OR (95% CI)	1.27 (1.14 to 1.43)	1.33 (1.16 to 1.53)		1.37 (1.15 to 1.63)	1.36 (1.14 to 1.62)	0.79 (0.43 to 1.45)
	*P*-value	**3.02E-05**	**4.36E-05**		**4.69E-04**	**5.21E-04**	0.44
rs181359	OR (95% CI)	1.16 (1.04 to 1.30)	1.25 (1.03 to 1.52)	0.92 (0.77 to 1.11)		0.17 (0.01 to 3.89)	0.98 (0.83 to 1.14)
	*P*-value	8.88E-03	**0.02**	0.39		0.27	0.76
rs5754217	OR (95% CI)	1.17 (1.04 to 1.31)	1.3 (1.08 to 1.57)	0.93 (0.77 to 1.11)	0.94 (0.77 to 1.11)		0.98 (0.84 to 1.14)
	*P*-value	**7.21E-03**	**6.75E-03**	0.41	0.20		0.79
rs2298428	OR (95% CI)	1.27 (1.14 to 1.41)	1.33 (1.18 to 1.51)	1.61 (0.87 to 2.95)	1.31 (1.13 to 1.53)	1.32 (1.13 to 1.53)	
	*P*-value	**1.61E-05**	**7.08E-06**	0.13	**5.14E-04**	**3.32E-04**	

^a^CI, Confidence interval; OR, Odds ratio; SNP, Single-nucleotide polymorphism. Independence tests were performed using the two genome-wide association datasets. Conditional *P*-values <0.05 are shown in bold.

### Replication on single-nucleotide polymorphism rs2298428

Replication for SNP rs2298428 was performed by using a TaqMan SNP genotyping method (Life Technologies) on the Hong Kong cohort independent from Hong Kong samples genotyped in the GWAS stage; samples collected from Bangkok, Thailand; and samples from Anhui, China, which were independent from the Anhui GWAS cohort. As shown in Table 5, replications in different cohorts showed consistent results as those from the discovery stage (Table 1). The joint analysis of association, taking into account the effect of SNP differences among cohorts from Hong Kong, Anhui and Thailand from both discovery stage and replication stage, was conducted using the Cochran-Mantel-Haenszel test. SNP rs2298428 showed stronger evidence of association with SLE (*P* =1.31E-11), and testing of between-population heterogeneity of odds ratios by the Breslow-Day test did not show significant differences among the cohorts (*P*_het =0.26).

**Table 5:** **The expression quantitative trait loci evidence on the single-nucleotide polymorphism rs2298428 on systemic lupus erythematosus and other immune-related diseases** ^**a**^

**SNP**	***UBE2L3***									
	**HapMap_LCL**								**B cell**	**Monocyte**
	**CEU**	**CHB**	**GIH**	**JPT**	**LWK**	**MEX**	**MKK**	**YRI**		
rs2298428	0.0012	0.0136	0.0158	9.4E-06	–	0.0047	–	–	1.3E-05	9.6E-15

^a^AH, Anhui; CEU, Utah residents with ancestry from northern and western Europe; CHB, Han Chinese in Bejing, China; GIH, Gujarati Indian from Houston, Texas;JPT,Japanese in Tokyo, Japan; LWK,Luhya in Webuye, Kenya; MEX,Mexican ancestry in Los Angeles, California; MKK,Maasai in Kinyawa, Kenya; YRI,Yoruba in Ibadan, Nigeria; LCL, Lymphoblastoid cell line; SNP, Single-nucleotide polymorphism.

### Expression quantitative trait loci in this region

Expression quantitative trait loci (eQTL) associations between SNP rs2298428 and *UBE2L3* and other genes in this region were closely examined. Two datasets, from Stranger *et al*. [28] and Fairfax *et al*. [29], were investigated. In the first study, the researchers examined the correlation of SNPs to gene expression using the lymphoblastoid cell lines (LCLs) of 726 individuals in 8 cohorts from the HapMap3 project. In the second study, the investigators assessed the correlation of SNPs to gene expression in a cell-specific manner using 288 paired, purified primary monocytes and B cells from Caucasians. As shown in Table 5, the genotype of SNP rs2298428 correlated with expression of *UBE2L3* in five different populations. (The other three cohorts did not show significant correlation, possibly due to the low frequency of the alternative allele, thus lower power.) The genotypes of the SNP also significantly correlated with the expression of *UBE2L3* in B cells and monocytes. The results consistently demonstrated that the risk allele T from rs2298428 is correlated with higher expression of *UBE2L3*.

The gene expression pattern of *UBE2L3* was also examined using a publicly available database, NextBio [30] (Additional file 3). Five independent studies reported increased expression of *UBE2L3* from patients with lupus compared with healthy controls in different cell lines. In addition, *UBE2L3* was found to have increased expression in a number of other autoimmune diseases, including CD, T1D, SS, PS and PA, using the same database, indicating that *UBE2L3* might be the key player in disease association of this region.

## Discussion

In this study, through meta-analysis of two existing GWAS on Han Chinese populations with a total number of 1,659 cases and 3,398 controls matched geographically, we identified SNP rs2298428 as the SNP with the highest association with SLE in the 22q11.21 region (*P*_meta =2.70E-09). The association of rs2298428 was further supported by replication in three cohorts from Hong Kong, Anhui and Thailand, and the results improved by two orders of magnitude after joint analysis from the discovery stage and the replication stage (*P*_all =1.31E-11, odds ratio =1.23).

Many GWAS hits were aggregated in this region for different autoimmune diseases, and here we tried to find out whether all the reported SNPs (six SNPs included) were linked to the same causal variant or whether they were derived from independent signals. All of the SNPs showed strong evidence of association with SLE in the current investigation with *P*_meta <7.27E-05. Stepwise logistic regression and conditional logistic regression were performed to examine the independence of these SNPs. The results supported the notion that rs2298428 exhibited the strongest association with SLE. According to the LD pattern, SNP rs463426 is relatively independent from the other five SNPs (Figure 1). However, we were unable to find evidence of independence based on the presently reported results (Figure 2). This might be due to the fact that the exploration of independently contributing variants from this region is based mainly on the meta-analysis data, which might not have enough power to detect multiple independent signals. The five SNPs reported for different autoimmune diseases are located in the same LD block in Chinese population, and their association may be derived from the same casual variant. These cross-phenotype associations in this region highlighted the shared genetic involvement in autoimmune diseases.

In addition, to identify how this region might influence susceptibility to SLE and other autoimmune diseases, we investigated the potential biological function of the gene. eQTL analysis is an important approach in detecting functional mechanisms underlying association by testing whether identified variants may lead to variations in mRNA expression of nearby genes. Using publicly available eQTL datasets, the SNPs in the 22q11.21 region were analyzed. All the results pointed to increased expression of *UBE2L3* as the mechanism for association with SLE. *UBE2L3* encodes a member of the E2 ubiquitin-conjugating enzyme family. This enzyme was demonstrated to participate in the ubiquitination of p53, c-Fos and the nuclear factor κB precursor p105 *in vitro* [31,32]. There is also evidence showing the interaction between UBE2L3 and RNF125 [33]. RNF125 is reported as a negative regulator of type I interferon (IFN) signaling. It is well known that patients with SLE have elevated serum levels of type I IFN [34] and that these increased levels correlate with disease activity and severity [35]. Among numerous immunologic alterations present in patients with lupus, the type I IFN system is thought to play a pivotal role in pathogenesis [36-38], which points to a possible role of *UBE2L3*. However, the exact mechanism of *UBE2L3* is still not fully understood.

## Conclusions

Focusing on the SNPs in 22q11.21 region with strong evidence of being associated with SLE in previous work, we have identified one more novel susceptibility variant showing the most significant genetic contribution for SLE via meta-analysis and further replication in independent cohorts. The putative susceptibility gene, *UBE2L3*, is suggested to be related to the type I IFN signaling pathway in SLE pathogenesis. Our findings may shed light on the shared biological mechanisms between different diseases with immunological components.

## Additional files

Additional file 1:
**Detailed information of the sample included in this study.**
Additional file 2:
**Meta-analysis results on the 22q11.21 region with** ***P*** **_meta < 0.0001 after imputation.**
Additional file 3:
**The public available expression data showing the higher expression of** ***UBE2L3*** **in SLE patients.**

## Abbreviations

AA: Amino acid
CD: Crohn’s disease
CeD: Celiac disease
CI: Confidence interval
eQTL: Expression quantitative trait loci
GWAS: Genome-wide association studies
HWE: Hardy-Weinberg equilibrium
IBD: Inflammatory bowel disease
IFN: Interferon
LCL: Lymphoblastoid cell line
LD: Linkage disequilibrium
OR: Odds ratio
PS: Psoriasis
RA: Rheumatoid arthritis
SLE: Systemic lupus erythematosus
SNP: Single-nucleotide polymorphism
SSc: Systemic sclerosis
