# Supplementary material for: Meta-analysis of two Chinese populations identifies an autoimmune disease risk allele in 22q11.21 as associated with systemic lupus erythematosus
Source: Arthritis Res Ther. 2015 Mar 20;17(1):67. doi: 10.1186/s13075-015-0577-6 (PMC4404227; doi:10.1186/s13075-015-0577-6)
Supplement: Additional file 1: — Detailed information of the sample included in this study. [file 13075_2015_577_MOESM1_ESM.docx]

| **The detailed information of the sample included in this study.** | | | | | | |
| --- | --- | --- | --- | --- | --- | --- |
| Sample | Discover Panel | | Replication Panel | | | SUM |
|  | HK_GWAS | AH_GWAS | HK_REP | AH_REP | TH_REP |  |
| CASE | 612 | 1047 | 793 | 1359 | 460 | 4271 |
| CONTROL | 2193 | 1205 | - | 1358 | 965 | 5721 |
