# Supplementary material for: Meta-analysis of two Chinese populations identifies an autoimmune disease risk allele in 22q11.21 as associated with systemic lupus erythematosus
Source: Arthritis Res Ther. 2015 Mar 20;17(1):67. doi: 10.1186/s13075-015-0577-6 (PMC4404227; doi:10.1186/s13075-015-0577-6)
Supplement: Additional file 2: — Meta-analysis results on the 22q11.21 region with P _meta < 0.0001 after imputation. [file 13075_2015_577_MOESM2_ESM.docx]

**Meta-analysis results on the 22q11.21 region with P_meta<0.0001 after imputation.**

| G/I | RSID | Chr_POS | A1/A2 | HKGWAS | | | |  | AHGWAS | | | |  | META-ANALYSIS | | |
| --- | --- | --- | --- | --- | --- | --- | --- | --- | --- | --- | --- | --- | --- | --- | --- | --- |
|  |  |  |  | INFO_HK | OR_HK | P_HK | SE_HK |  | INFO_AH | OR_AH | P_AH | SE_AH |  | P-value | Direction | HetPVal |
| I | rs142577326 | 21765202 | A/G | 0.58 | 0.90 | 0.04 | 0.09 |  | 0.57 | 0.84 | 1.05E-04 | 0.08 |  | 4.70E-05 | ++ | 0.23 |
| I | rs458361 | 21798351 | C/G | 0.98 | 0.88 | 0.05 | 0.07 |  | 0.97 | 0.77 | 8.27E-06 | 0.06 |  | 4.69E-06 | -- | 0.12 |
| G | rs465500 | 21798907 | A/G | 1.00 | 0.90 | 0.09 | 0.07 |  | 1.00 | 0.78 | 2.98E-05 | 0.06 |  | 2.55E-05 | ++ | 0.12 |
| I | rs464694 | 21801489 | A/C | 0.98 | 0.89 | 0.06 | 0.07 |  | 0.98 | 0.77 | 1.33E-05 | 0.06 |  | 7.31E-06 | -- | 0.13 |
| I | rs3747086 | 21802961 | T/G | 0.87 | 1.16 | 0.02 | 0.07 |  | 0.88 | 1.27 | 3.12E-05 | 0.06 |  | 2.75E-06 | -- | 0.31 |
| G | rs460106 | 21806401 | T/C | 1.00 | 0.90 | 0.09 | 0.07 |  | 1.00 | 0.78 | 4.42E-05 | 0.06 |  | 3.12E-05 | ++ | 0.14 |
| I | rs34043275 | 21809133 | A/G | 0.87 | 1.15 | 0.02 | 0.07 |  | 0.88 | 1.28 | 1.09E-05 | 0.06 |  | 1.48E-06 | ++ | 0.22 |
| **G** | **rs463426** | **21809185** | **T/C** | **1.00** | **0.89** | **0.08** | **0.07** |  | **1.00** | **0.78** | **3.62E-05** | **0.06** |  | **2.50E-05** | **++** | **0.14** |
| I | rs455546 | 21809438 | T/G | 1.00 | 0.89 | 0.06 | 0.07 |  | 1.00 | 0.78 | 4.06E-05 | 0.06 |  | 2.01E-05 | -- | 0.17 |
| I | rs5753607 | 21812167 | A/G | 0.85 | 1.15 | 0.02 | 0.07 |  | 0.86 | 1.28 | 8.05E-06 | 0.07 |  | 1.56E-06 | ++ | 0.19 |
| I | rs114587380 | 21818481 | T/G | 0.85 | 0.90 | 0.08 | 0.07 |  | 0.84 | 0.81 | 9.05E-05 | 0.07 |  | 5.19E-05 | ++ | 0.18 |
| I | rs74345854 | 21819510 | A/G | 0.70 | 0.86 | 0.01 | 0.08 |  | 0.69 | 0.82 | 6.64E-05 | 0.07 |  | 2.15E-06 | ++ | 0.54 |
| I | rs434916 | 21829215 | T/C | 0.75 | 0.89 | 0.03 | 0.08 |  | 0.74 | 0.81 | 2.71E-05 | 0.07 |  | 8.32E-06 | -- | 0.20 |
| I | rs74875706 | 21832470 | A/C | 0.75 | 0.88 | 0.03 | 0.08 |  | 0.74 | 0.80 | 2.04E-05 | 0.07 |  | 4.79E-06 | ++ | 0.21 |
| I | rs183606830 | 21837221 | T/C | 0.55 | 0.87 | 4.81E-03 | 0.09 |  | 0.55 | 0.87 | 2.26E-03 | 0.08 |  | 5.05E-05 | ++ | 0.99 |
| I | rs5753762 | 21840280 | T/C | 0.69 | 1.15 | 0.01 | 0.08 |  | 0.68 | 1.28 | 1.35E-06 | 0.07 |  | 1.51E-07 | ++ | 0.17 |
| I | rs399557 | 21840326 | T/C | 0.74 | 0.87 | 0.02 | 0.08 |  | 0.73 | 0.81 | 2.29E-05 | 0.07 |  | 2.70E-06 | ++ | 0.28 |
| I | rs74193520 | 21840405 | A/G | 0.69 | 1.15 | 0.01 | 0.08 |  | 0.68 | 1.28 | 1.32E-06 | 0.07 |  | 1.47E-07 | ++ | 0.16 |
| I | rs116999122 | 21842813 | T/C | 0.74 | 0.88 | 0.02 | 0.08 |  | 0.73 | 0.81 | 2.61E-05 | 0.07 |  | 4.14E-06 | -- | 0.26 |
| I | rs8139142 | 21910280 | T/C | 0.76 | 1.14 | 0.02 | 0.08 |  | 0.77 | 1.30 | 2.18E-06 | 0.07 |  | 5.76E-07 | -- | 0.13 |
| I | rs5754075 | 21911220 | A/G | 0.73 | 1.18 | 4.40E-03 | 0.08 |  | 0.74 | 1.32 | 7.97E-07 | 0.08 |  | 4.26E-08 | ++ | 0.18 |
| I | rs4821059 | 21911333 | A/T | 0.78 | 1.14 | 0.03 | 0.08 |  | 0.78 | 1.31 | 2.64E-06 | 0.07 |  | 1.05E-06 | -- | 0.11 |
| I | rs12168746 | 21912216 | A/C | 0.80 | 1.15 | 0.01 | 0.07 |  | 0.81 | 1.33 | 9.80E-07 | 0.07 |  | 1.67E-07 | ++ | 0.13 |
| I | rs367826 | 21912337 | T/C | 0.76 | 0.88 | 0.03 | 0.07 |  | 0.79 | 0.82 | 2.26E-04 | 0.07 |  | 3.35E-05 | -- | 0.36 |
| I | rs5754100 | 21916166 | T/C | 0.92 | 1.21 | 1.94E-03 | 0.07 |  | 0.93 | 1.35 | 4.30E-07 | 0.06 |  | 6.98E-09 | -- | 0.26 |
| I | rs5754102 | 21916272 | A/C | 0.84 | 1.15 | 0.02 | 0.07 |  | 0.85 | 1.35 | 4.97E-07 | 0.07 |  | 1.54E-07 | ++ | 0.10 |
| **G** | **rs131654** | **21917190** | **T/G** | **1.00** | **1.08** | **0.24** | **0.07** |  | **1.00** | **1.29** | **1.60E-05** | **0.06** |  | **7.27E-05** | **++** | **0.04** |
| I | rs131656 | 21917450 | A/G | 0.99 | 1.12 | 0.07 | 0.07 |  | 0.99 | 1.32 | 3.36E-06 | 0.06 |  | 3.58E-06 | ++ | 0.08 |
| I | rs5998509 | 21917479 | T/C | 0.96 | 1.22 | 1.75E-03 | 0.07 |  | 0.96 | 1.35 | 8.13E-07 | 0.06 |  | 1.01E-08 | ++ | 0.32 |
| I | rs131657 | 21917550 | A/T | 0.94 | 1.13 | 0.05 | 0.07 |  | 0.95 | 1.31 | 4.88E-06 | 0.06 |  | 2.73E-06 | ++ | 0.11 |
| I | rs131658 | 21917626 | C/G | 0.99 | 1.12 | 0.08 | 0.07 |  | 0.99 | 1.33 | 2.64E-06 | 0.06 |  | 3.86E-06 | -- | 0.06 |
| I | rs131660 | 21917757 | A/G | 0.91 | 1.10 | 0.12 | 0.07 |  | 0.92 | 1.33 | 8.93E-07 | 0.06 |  | 3.97E-06 | ++ | 0.03 |
| I | rs138665726 | 21917859 | A/C | 0.95 | 1.22 | 1.54E-03 | 0.07 |  | 0.96 | 1.36 | 4.38E-07 | 0.06 |  | 5.23E-09 | -- | 0.29 |
| I | rs140488 | 21919671 | A/G | 0.99 | 1.12 | 0.07 | 0.07 |  | 0.99 | 1.33 | 2.07E-06 | 0.06 |  | 2.65E-06 | -- | 0.06 |
| I | rs131664 | 21920403 | A/G | 0.99 | 1.12 | 0.07 | 0.07 |  | 0.99 | 1.33 | 2.10E-06 | 0.06 |  | 2.68E-06 | ++ | 0.06 |
| I | rs59391722 | 21920817 | C/G | 0.97 | 1.22 | 1.67E-03 | 0.07 |  | 0.98 | 1.37 | 2.75E-07 | 0.06 |  | 3.90E-09 | ++ | 0.26 |
| I | rs131665 | 21920903 | A/G | 0.99 | 1.12 | 0.07 | 0.07 |  | 0.99 | 1.33 | 2.00E-06 | 0.06 |  | 2.54E-06 | -- | 0.06 |
| I | rs140489 | 21921294 | A/G | 1.00 | 1.12 | 0.09 | 0.07 |  | 1.00 | 1.33 | 1.57E-06 | 0.06 |  | 2.80E-06 | ++ | 0.05 |
| I | rs140490 | 21921686 | T/G | 1.00 | 1.12 | 0.09 | 0.07 |  | 1.00 | 1.33 | 1.57E-06 | 0.06 |  | 2.77E-06 | ++ | 0.05 |
| I | rs140491 | 21922364 | T/C | 0.99 | 1.12 | 0.07 | 0.07 |  | 0.99 | 1.33 | 1.91E-06 | 0.06 |  | 2.47E-06 | -- | 0.06 |
| I | rs11089620 | 21922456 | C/G | 0.97 | 1.22 | 1.67E-03 | 0.07 |  | 0.97 | 1.36 | 3.31E-07 | 0.06 |  | 4.57E-09 | -- | 0.27 |
| **I** | **rs2266959** | **21922904** | **T/G** | **0.97** | **1.22** | **1.66E-03** | **0.07** |  | **0.97** | **1.37** | **3.22E-07** | **0.06** |  | **4.41E-09** | **++** | **0.27** |
| I | rs140492 | 21923144 | A/C | 1.00 | 1.12 | 0.08 | 0.07 |  | 1.00 | 1.33 | 1.57E-06 | 0.06 |  | 2.74E-06 | -- | 0.05 |
| I | rs2256609 | 21925017 | A/G | 0.97 | 1.22 | 1.62E-03 | 0.07 |  | 0.97 | 1.37 | 2.56E-07 | 0.06 |  | 3.55E-09 | -- | 0.26 |
| I | rs140496 | 21926456 | A/G | 0.97 | 1.11 | 0.11 | 0.07 |  | 0.96 | 1.32 | 2.29E-06 | 0.06 |  | 5.64E-06 | -- | 0.05 |
| I | rs140498 | 21927064 | A/G | 0.99 | 1.12 | 0.07 | 0.07 |  | 0.99 | 1.33 | 1.86E-06 | 0.06 |  | 2.37E-06 | ++ | 0.06 |
| I | rs140499 | 21927231 | T/C | 0.99 | 1.12 | 0.07 | 0.07 |  | 0.99 | 1.33 | 1.97E-06 | 0.06 |  | 2.41E-06 | ++ | 0.06 |
| I | rs2266961 | 21928597 | C/G | 0.97 | 1.22 | 1.62E-03 | 0.07 |  | 0.97 | 1.37 | 2.59E-07 | 0.06 |  | 3.57E-09 | -- | 0.26 |
| **I** | **rs181359** | **21928641** | **A/G** | **0.99** | **1.12** | **0.07** | **0.07** |  | **0.99** | **1.33** | **1.86E-06** | **0.06** |  | **2.36E-06** | **++** | **0.06** |
| I | rs181360 | 21928916 | T/G | 0.99 | 1.12 | 0.07 | 0.07 |  | 0.99 | 1.33 | 1.86E-06 | 0.06 |  | 2.36E-06 | -- | 0.06 |
| I | rs181361 | 21929566 | A/T | 0.91 | 1.09 | 0.18 | 0.07 |  | 0.92 | 1.33 | 5.74E-07 | 0.06 |  | 5.46E-06 | -- | 0.02 |
| I | rs73166619 | 21930093 | T/C | 0.97 | 1.22 | 1.61E-03 | 0.07 |  | 0.97 | 1.37 | 2.59E-07 | 0.06 |  | 3.56E-09 | ++ | 0.26 |
| I | rs5754166 | 21930777 | T/C | 0.97 | 1.22 | 1.61E-03 | 0.07 |  | 0.97 | 1.37 | 3.02E-07 | 0.06 |  | 4.05E-09 | ++ | 0.26 |
| I | rs181362 | 21932068 | T/C | 1.00 | 1.12 | 0.09 | 0.07 |  | 1.00 | 1.34 | 1.47E-06 | 0.06 |  | 2.66E-06 | ++ | 0.05 |
| I | rs181363 | 21932264 | A/G | 1.00 | 1.12 | 0.09 | 0.07 |  | 1.00 | 1.34 | 1.48E-06 | 0.06 |  | 2.67E-06 | -- | 0.05 |
| I | rs5754177 | 21933428 | T/C | 0.89 | 1.20 | 2.57E-03 | 0.07 |  | 0.90 | 1.33 | 9.34E-07 | 0.06 |  | 1.93E-08 | ++ | 0.28 |
| I | rs181366 | 21933780 | T/C | 0.99 | 1.12 | 0.07 | 0.07 |  | 0.99 | 1.33 | 2.03E-06 | 0.06 |  | 2.55E-06 | ++ | 0.06 |
| I | rs66534072 | 21936152 | C/G | 1.00 | 1.12 | 0.09 | 0.07 |  | 1.00 | 1.34 | 1.12E-06 | 0.06 |  | 2.23E-06 | -- | 0.05 |
| I | rs374387 | 21936835 | A/C | 0.98 | 1.08 | 0.26 | 0.07 |  | 0.98 | 1.30 | 1.29E-05 | 0.06 |  | 7.14E-05 | ++ | 0.04 |
| I | rs5749485 | 21938224 | A/C | 1.00 | 1.12 | 0.09 | 0.07 |  | 1.00 | 1.34 | 1.01E-06 | 0.06 |  | 2.06E-06 | -- | 0.04 |
| I | rs5998576 | 21938590 | T/C | 0.98 | 1.12 | 0.07 | 0.07 |  | 0.98 | 1.34 | 7.00E-07 | 0.06 |  | 1.09E-06 | ++ | 0.05 |
| I | rs73166622 | 21938863 | C/G | 0.99 | 1.12 | 0.08 | 0.07 |  | 0.98 | 1.33 | 1.89E-06 | 0.06 |  | 3.07E-06 | ++ | 0.06 |
| **G** | **rs5754217** | **21939675** | **T/G** | **1.00** | **1.12** | **0.09** | **0.07** |  | **1.00** | **1.34** | **1.04E-06** | **0.06** |  | **2.07E-06** | **++** | **0.04** |
| I | rs5749493 | 21939687 | A/C | 1.00 | 1.12 | 0.09 | 0.07 |  | 1.00 | 1.34 | 1.04E-06 | 0.06 |  | 2.10E-06 | ++ | 0.04 |
| I | rs4820091 | 21940189 | T/G | 1.00 | 1.12 | 0.09 | 0.07 |  | 1.00 | 1.34 | 1.04E-06 | 0.06 |  | 2.10E-06 | -- | 0.04 |
| I | rs5749495 | 21940310 | A/G | 0.98 | 1.22 | 1.70E-03 | 0.07 |  | 0.98 | 1.37 | 2.48E-07 | 0.06 |  | 3.66E-09 | -- | 0.25 |
| I | rs12484550 | 21941915 | T/C | 0.97 | 1.20 | 3.48E-03 | 0.07 |  | 0.97 | 1.37 | 2.24E-07 | 0.06 |  | 8.27E-09 | ++ | 0.18 |
| I | rs5998599 | 21941981 | A/G | 0.99 | 1.12 | 0.07 | 0.07 |  | 0.99 | 1.33 | 1.47E-06 | 0.06 |  | 2.18E-06 | -- | 0.06 |
| I | rs9621715 | 21942007 | A/G | 0.97 | 1.20 | 3.48E-03 | 0.07 |  | 0.97 | 1.37 | 2.24E-07 | 0.06 |  | 8.26E-09 | ++ | 0.18 |
| I | rs5754234 | 21942978 | A/T | 0.97 | 1.20 | 3.48E-03 | 0.07 |  | 0.97 | 1.37 | 2.25E-07 | 0.06 |  | 8.31E-09 | -- | 0.18 |
| I | rs5754238 | 21943664 | C/G | 0.93 | 1.11 | 0.10 | 0.07 |  | 0.94 | 1.33 | 9.29E-07 | 0.06 |  | 2.76E-06 | -- | 0.04 |
| I | rs1034329 | 21943938 | T/C | 0.99 | 1.12 | 0.07 | 0.07 |  | 0.99 | 1.33 | 1.56E-06 | 0.06 |  | 2.12E-06 | -- | 0.06 |
| I | rs2283789 | 21944478 | T/G | 0.99 | 1.12 | 0.07 | 0.07 |  | 0.99 | 1.33 | 1.56E-06 | 0.06 |  | 2.12E-06 | -- | 0.06 |
| I | rs5749502 | 21945096 | A/T | 0.97 | 1.20 | 3.45E-03 | 0.07 |  | 0.97 | 1.37 | 2.25E-07 | 0.06 |  | 8.21E-09 | ++ | 0.18 |
| I | rs5998619 | 21945851 | A/G | 0.99 | 1.13 | 0.07 | 0.07 |  | 0.99 | 1.33 | 2.16E-06 | 0.06 |  | 2.56E-06 | ++ | 0.07 |
| I | rs73166630 | 21945978 | A/G | 0.96 | 1.20 | 2.90E-03 | 0.07 |  | 0.96 | 1.36 | 3.29E-07 | 0.06 |  | 8.86E-09 | ++ | 0.22 |
| I | rs73166632 | 21946173 | A/G | 0.99 | 1.12 | 0.07 | 0.07 |  | 0.99 | 1.33 | 1.58E-06 | 0.06 |  | 2.11E-06 | -- | 0.06 |
| I | rs2266963 | 21947467 | C/G | 0.97 | 1.20 | 3.38E-03 | 0.07 |  | 0.97 | 1.37 | 2.27E-07 | 0.06 |  | 8.06E-09 | -- | 0.19 |
| I | rs2070512 | 21949411 | A/C | 1.00 | 1.12 | 0.08 | 0.07 |  | 1.00 | 1.34 | 1.24E-06 | 0.06 |  | 2.05E-06 | -- | 0.05 |
| I | rs4821104 | 21950405 | A/G | 0.97 | 1.21 | 2.88E-03 | 0.07 |  | 0.97 | 1.37 | 2.38E-07 | 0.06 |  | 6.74E-09 | -- | 0.20 |
| I | rs5754295 | 21951740 | A/C | 0.99 | 1.13 | 0.05 | 0.07 |  | 0.99 | 1.32 | 3.06E-06 | 0.06 |  | 2.30E-06 | ++ | 0.09 |
| I | rs5994638 | 21953276 | A/G | 0.99 | 1.13 | 0.06 | 0.07 |  | 0.99 | 1.33 | 1.93E-06 | 0.06 |  | 1.98E-06 | -- | 0.07 |
| I | rs5998644 | 21953288 | T/C | 0.99 | 1.13 | 0.06 | 0.07 |  | 0.99 | 1.33 | 2.13E-06 | 0.06 |  | 2.17E-06 | -- | 0.07 |
| I | rs2283790 | 21956653 | A/G | 1.00 | 1.13 | 0.05 | 0.07 |  | 0.99 | 1.32 | 3.65E-06 | 0.06 |  | 2.42E-06 | -- | 0.10 |
| I | rs5754323 | 21957992 | T/C | 0.99 | 1.13 | 0.06 | 0.07 |  | 0.99 | 1.33 | 2.35E-06 | 0.06 |  | 2.13E-06 | -- | 0.08 |
| I | rs2266964 | 21958304 | A/G | 0.99 | 1.13 | 0.06 | 0.07 |  | 0.99 | 1.33 | 1.62E-06 | 0.06 |  | 1.81E-06 | -- | 0.07 |
| I | rs11089629 | 21958872 | T/G | 0.99 | 1.13 | 0.06 | 0.07 |  | 0.99 | 1.33 | 1.73E-06 | 0.06 |  | 1.89E-06 | -- | 0.07 |
| I | rs4821108 | 21959038 | C/G | 1.00 | 1.13 | 0.05 | 0.07 |  | 1.00 | 1.33 | 2.51E-06 | 0.06 |  | 1.89E-06 | ++ | 0.09 |
| I | rs5754344 | 21963786 | A/G | 0.98 | 1.23 | 1.02E-03 | 0.07 |  | 0.98 | 1.36 | 4.90E-07 | 0.06 |  | 3.60E-09 | -- | 0.34 |
| G | rs4821112 | 21964761 | A/G | 1.00 | 1.14 | 0.05 | 0.07 |  | 1.00 | 1.32 | 4.79E-06 | 0.06 |  | 2.64E-06 | ++ | 0.11 |
| I | rs5754352 | 21964951 | T/C | 1.00 | 1.14 | 0.05 | 0.07 |  | 1.00 | 1.32 | 4.39E-06 | 0.06 |  | 2.40E-06 | ++ | 0.11 |
| I | rs5998672 | 21966442 | A/G | 0.99 | 1.12 | 0.08 | 0.07 |  | 0.99 | 1.32 | 4.38E-06 | 0.06 |  | 4.94E-06 | ++ | 0.08 |
| I | rs12168958 | 21966804 | T/C | 0.89 | 1.16 | 0.02 | 0.07 |  | 0.90 | 1.33 | 1.11E-06 | 0.06 |  | 2.28E-07 | ++ | 0.12 |
| I | rs12169001 | 21966833 | T/C | 0.95 | 1.13 | 0.04 | 0.07 |  | 0.96 | 1.32 | 4.10E-06 | 0.06 |  | 2.16E-06 | ++ | 0.11 |
| I | rs738127 | 21968221 | A/G | 1.00 | 1.14 | 0.05 | 0.07 |  | 1.00 | 1.32 | 4.36E-06 | 0.06 |  | 2.38E-06 | ++ | 0.11 |
| I | rs8137950 | 21969640 | T/C | 0.95 | 1.13 | 0.05 | 0.07 |  | 0.96 | 1.31 | 4.91E-06 | 0.06 |  | 2.92E-06 | -- | 0.11 |
| I | rs4821114 | 21970810 | C/G | 0.98 | 1.24 | 6.49E-04 | 0.07 |  | 0.98 | 1.35 | 9.00E-07 | 0.06 |  | 3.56E-09 | ++ | 0.44 |
| I | rs738128 | 21971010 | A/G | 0.89 | 1.10 | 0.11 | 0.07 |  | 0.89 | 1.34 | 2.76E-07 | 0.06 |  | 1.36E-06 | ++ | 0.02 |
| I | rs738129 | 21971041 | T/C | 0.82 | 1.09 | 0.12 | 0.07 |  | 0.82 | 1.32 | 8.56E-07 | 0.07 |  | 3.62E-06 | ++ | 0.03 |
| I | rs8139079 | 21971632 | T/G | 1.00 | 1.14 | 0.05 | 0.07 |  | 1.00 | 1.32 | 4.32E-06 | 0.06 |  | 2.33E-06 | -- | 0.11 |
| I | rs2876971 | 21972401 | T/G | 0.99 | 1.15 | 0.03 | 0.07 |  | 0.98 | 1.32 | 4.60E-06 | 0.06 |  | 1.57E-06 | -- | 0.14 |
| I | rs4821116 | 21973319 | T/C | 0.98 | 1.23 | 8.99E-04 | 0.07 |  | 0.98 | 1.36 | 7.09E-07 | 0.06 |  | 4.20E-09 | ++ | 0.38 |
| I | rs5754387 | 21974703 | C/G | 0.85 | 1.12 | 0.07 | 0.07 |  | 0.86 | 1.27 | 1.88E-05 | 0.06 |  | 1.32E-05 | ++ | 0.14 |
| I | rs7444 | 21976934 | T/C | 0.99 | 1.13 | 0.06 | 0.07 |  | 0.99 | 1.32 | 2.89E-06 | 0.06 |  | 2.29E-06 | -- | 0.08 |
| I | rs7445 | 21977047 | T/C | 0.98 | 1.14 | 0.04 | 0.07 |  | 0.98 | 1.32 | 4.78E-06 | 0.06 |  | 2.19E-06 | ++ | 0.12 |
| I | rs11089637 | 21979096 | T/C | 0.98 | 1.13 | 0.05 | 0.07 |  | 0.98 | 1.32 | 2.57E-06 | 0.06 |  | 2.07E-06 | -- | 0.08 |
| I | rs4821124 | 21979289 | T/C | 0.98 | 1.23 | 8.88E-04 | 0.07 |  | 0.98 | 1.36 | 6.01E-07 | 0.06 |  | 3.58E-09 | -- | 0.37 |
| I | rs12158299 | 21979584 | T/C | 1.00 | 1.14 | 0.05 | 0.07 |  | 1.00 | 1.32 | 4.11E-06 | 0.06 |  | 2.21E-06 | ++ | 0.11 |
| I | rs9619386 | 21979938 | T/C | 0.98 | 1.15 | 0.03 | 0.07 |  | 0.98 | 1.30 | 1.03E-05 | 0.06 |  | 2.74E-06 | ++ | 0.17 |
| I | rs5754422 | 21980257 | A/G | 0.98 | 1.15 | 0.03 | 0.07 |  | 0.98 | 1.30 | 1.09E-05 | 0.06 |  | 2.87E-06 | -- | 0.17 |
| I | rs5754426 | 21980638 | T/C | 0.98 | 1.15 | 0.03 | 0.07 |  | 0.98 | 1.30 | 9.64E-06 | 0.06 |  | 2.56E-06 | -- | 0.17 |
| I | rs4821130 | 21980894 | T/C | 0.98 | 1.15 | 0.03 | 0.07 |  | 0.98 | 1.30 | 9.43E-06 | 0.06 |  | 2.50E-06 | ++ | 0.17 |
| I | rs1811069 | 21982054 | T/G | 0.94 | 1.12 | 0.06 | 0.07 |  | 0.94 | 1.34 | 9.45E-07 | 0.06 |  | 1.23E-06 | -- | 0.06 |
| I | rs878825 | 21982249 | T/C | 0.98 | 1.13 | 0.05 | 0.07 |  | 0.98 | 1.33 | 2.15E-06 | 0.06 |  | 1.64E-06 | -- | 0.08 |
| I | rs861857 | 21982340 | C/G | 0.97 | 1.12 | 0.07 | 0.07 |  | 0.96 | 1.31 | 5.97E-06 | 0.06 |  | 5.71E-06 | -- | 0.09 |
| **G** | **rs2298428** | **21982892** | **T/C** | **1.00** | **1.24** | **8.44E-04** | **0.07** |  | **1.00** | **1.37** | **4.72E-07** | **0.06** |  | **2.70E-09** | **++** | **0.36** |
| I | rs2298429 | 21983260 | A/G | 0.97 | 1.13 | 0.06 | 0.07 |  | 0.97 | 1.34 | 9.70E-07 | 0.06 |  | 1.24E-06 | -- | 0.06 |
| I | rs710177 | 21984205 | A/G | 0.94 | 1.11 | 0.09 | 0.07 |  | 0.94 | 1.32 | 1.92E-06 | 0.06 |  | 3.62E-06 | -- | 0.05 |
| I | rs3747093 | 21984379 | A/G | 0.94 | 1.11 | 0.09 | 0.07 |  | 0.94 | 1.33 | 8.69E-07 | 0.06 |  | 2.17E-06 | ++ | 0.04 |
| I | rs5754467 | 21985094 | A/G | 0.94 | 1.12 | 0.07 | 0.07 |  | 0.93 | 1.32 | 1.92E-06 | 0.06 |  | 2.34E-06 | -- | 0.07 |
| I | rs5754508 | 21999229 | C/G | 0.71 | 1.06 | 0.27 | 0.08 |  | 0.70 | 1.26 | 6.75E-06 | 0.07 |  | 7.24E-05 | -- | 0.02 |
