# Supplementary material for: Meta-analysis of two Chinese populations identifies an autoimmune disease risk allele in 22q11.21 as associated with systemic lupus erythematosus
Source: Arthritis Res Ther. 2015 Mar 20;17(1):67. doi: 10.1186/s13075-015-0577-6 (PMC4404227; doi:10.1186/s13075-015-0577-6)
Supplement: Additional file 3: — The public available expression data showing the higher expression of UBE2L3 in SLE patients. [file 13075_2015_577_MOESM3_ESM.docx]

**The public available expression data showing the higher expression of UBE2L3 in SLE patients**

| Gene Symbol | Monocyte | | | CD4+ T cell | | | Myeloid cell | | | CD19+ B cell | | |
| --- | --- | --- | --- | --- | --- | --- | --- | --- | --- | --- | --- | --- |
|  | GEO | Fold change | P value | GEO | Fold change | P value | GEO | Fold change | P value | GEO | Fold change | P value |
| UBE2L3 | GSE37356 | 1.23 | 0.0052 | GSE51997 | 1.62 | 0.006 | GSE10325 | 1.55 | 0.021 | GSE10325 | 1.38 | 0.0118 |
|  | GSE51997 | 1.38 | 0.0007 | - | - | - | - | - | - | - | - | - |
|  | GSE46923 | 1.67 | 0.006 | - | - | - | - | - | - | - | - | - |
|  | GSE38351 | 1.22 | 0.0303 | - | - | - | - | - | - | - | - | - |
